# 3-Chloro-*N*′-(2-chloro­benzyl­idene)benzohydrazide

**DOI:** 10.1107/S1600536811001656

**Published:** 2011-01-15

**Authors:** Yan Lei, Chuan Fu

**Affiliations:** a School of Chemistry & Environmental Engineering, Chongqing Three Gorges University, Chongqing 404000, People’s Republic of China

## Abstract

The title compound, C_14_H_10_Cl_2_N_2_O, was prepared from the reaction of 2-chloro­benzaldehyde with 3-chloro­benzo­hydrazide in methanol. The mol­ecule adopts an *E* configuration about the methyl­idene unit and the two aromatic rings form a dihedral angle of 13.8 (2)°. In the crystal, mol­ecules are linked *via* inter­molecular N—H⋯O and C—H⋯O hydrogen bonds, forming chains along the *c* axis.

## Related literature

For background to hydrazones, see: El-Asmy *et al.* (2010 ▶); El-Sherif (2009 ▶); Singh *et al.* (2009 ▶); El-Tabl *et al.* (2007 ▶); Lei (2011 ▶). For structures of hydrazone compounds, see: Qiao *et al.* (2010 ▶); Hussain *et al.* (2010 ▶); Han & Zhao (2010 ▶); Ahmad *et al.* (2010 ▶).
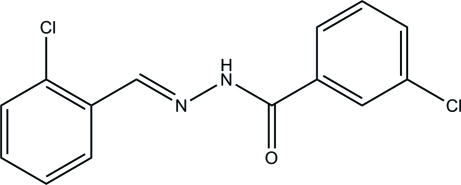


## Experimental

### 

#### Crystal data

C_14_H_10_Cl_2_N_2_O
*M* *_r_* = 293.14
Monoclinic, 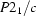

*a* = 13.106 (3) Å
*b* = 12.588 (3) Å
*c* = 8.347 (2) Å
β = 97.578 (2)°
*V* = 1365.0 (6) Å^3^
*Z* = 4
Mo *K*α radiation
μ = 0.47 mm^−1^
*T* = 298 K
0.32 × 0.30 × 0.30 mm

#### Data collection

Bruker SMART CCD area-detector diffractometer
Absorption correction: multi-scan (*SADABS*; Bruker, 2000 ▶) *T*_min_ = 0.865, *T*_max_ = 0.873
6893 measured reflections
2954 independent reflections
1936 reflections with *I* > 2σ(*I*)
*R*_int_ = 0.032

#### Refinement

*R*[*F* ^2^ > 2σ(*F* ^2^)] = 0.048
*wR*(*F* ^2^) = 0.130
*S* = 1.01
2954 reflections
175 parameters
1 restraint
H atoms treated by a mixture of independent and constrained refinement
Δρ_max_ = 0.26 e Å^−3^
Δρ_min_ = −0.48 e Å^−3^

### 

Data collection: *SMART* (Bruker, 2000 ▶); cell refinement: *SAINT* (Bruker, 2000 ▶); data reduction: *SAINT*; program(s) used to solve structure: *SHELXS97* (Sheldrick, 2008 ▶); program(s) used to refine structure: *SHELXL97* (Sheldrick, 2008 ▶); molecular graphics: *SHELXTL* (Sheldrick, 2008 ▶); software used to prepare material for publication: *SHELXTL*.

## Supplementary Material

Crystal structure: contains datablocks global, I. DOI: 10.1107/S1600536811001656/qm2001sup1.cif

Structure factors: contains datablocks I. DOI: 10.1107/S1600536811001656/qm2001Isup2.hkl

Additional supplementary materials:  crystallographic information; 3D view; checkCIF report

## Figures and Tables

**Table 1:** Hydrogen-bond geometry (Å, °)

*D*—H⋯*A*	*D*—H	H⋯*A*	*D*⋯*A*	*D*—H⋯*A*
N2—H2⋯O1^i^	0.90 (1)	1.98 (1)	2.854 (2)	164 (2)
C7—H7⋯O1^i^	0.93	2.51	3.254 (2)	137 (2)

Symmetry code: (i) 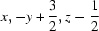.

**Fig. 1.**
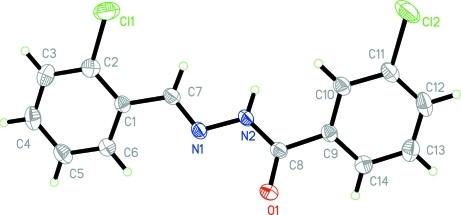
The molecular structure of the title compound with 30% probability displacement ellipsoids.

**Fig. 2.**
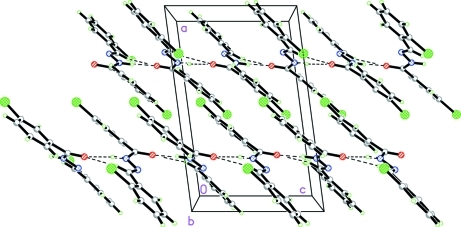
The molecular packing of the title compound. Hydrogen bonding is shown in dashed lines.

## supplementary crystallographic information

### Comment

In recent years, much effort has been devoted for developing the hydrazones, due to their biological properties, coordinative capability, and applications in analytical chemistry (El-Asmy *et al.*, 2010; El-Sherif, 2009; Singh *et al.*, 2009; El-Tabl *et al.*, 2007). Recently, a number of hydrazones have been prepared and investigated for their structures (Qiao *et al.*, 2010; Hussain *et al.*, 2010; Han & Zhao, 2010; Ahmad *et al.*, 2010; Lei, 2011). As a continuation of hydrazones, the author reports herein the title new compound.

The molecule of the title compound, Fig. 1, adopts an *E* configuration about the methylidene unit. The two aromatic rings form a dihedral angle of 13.8 (2)°. In the crystal, the molecules are linked *via* intermolecular N—H···O and C—H···O hydrogen bonds (Table 1), to form chains at the *c*-axis direction (Fig. 2).

### Experimental

3-Chlorobenzohydrazide (1 mmol, 0.170 g) was dissolved in methanol (50 ml), then 2-chlorobenzaldehyde (1 mmol, 0.140 g) was added into the solution. The reaction mixture was heated under reflux for 1 h and cooled to room temperature. Colourless needle-shaped crystals were formed by slow evaporation of the solvent for a week.

### Refinement

The amino H atom was located in a difference Fourier map and refined isotropically, with the N—H distance restrained to 0.90 (1) Å. Other H atoms were positioned geometrically and constrained to ride on their parent atoms, with C—H = 0.93 Å, and with *U*_iso_(H) = 1.2*U*_eq_(C).

### Crystal data

**Table:**

C_14_H_10_Cl_2_N_2_O	*F*(000) = 600
*M**_r_* = 293.14	*D*_x_ = 1.426 Mg m^−^^3^
Monoclinic, *P*2_1_/*c*	Mo *K*α radiation, λ = 0.71073 Å
*a* = 13.106 (3) Å	Cell parameters from 1672 reflections
*b* = 12.588 (3) Å	θ = 2.2–25.0°
*c* = 8.347 (2) Å	µ = 0.47 mm^−^^1^
β = 97.578 (2)°	*T* = 298 K
*V* = 1365.0 (6) Å^3^	Cut from needle, colourless
*Z* = 4	0.32 × 0.30 × 0.30 mm

### Data collection

**Table:**

Bruker SMART CCD area-detector diffractometer	2954 independent reflections
Radiation source: fine-focus sealed tube	1936 reflections with *I* > 2σ(*I*)
graphite	*R*_int_ = 0.032
ω scans	θ_max_ = 27.0°, θ_min_ = 2.3°
Absorption correction: multi-scan (*SADABS*; Bruker, 2000)	*h* = −16→8
*T*_min_ = 0.865, *T*_max_ = 0.873	*k* = −15→15
6893 measured reflections	*l* = −10→10

### Refinement

**Table:**

Refinement on *F*^2^	Primary atom site location: structure-invariant direct methods
Least-squares matrix: full	Secondary atom site location: difference Fourier map
*R*[*F*^2^ > 2σ(*F*^2^)] = 0.048	Hydrogen site location: inferred from neighbouring sites
*wR*(*F*^2^) = 0.130	H atoms treated by a mixture of independent and constrained refinement
*S* = 1.01	*w* = 1/[σ^2^(*F*_o_^2^) + (0.0682*P*)^2^] where *P* = (*F*_o_^2^ + 2*F*_c_^2^)/3
2954 reflections	(Δ/σ)_max_ < 0.001
175 parameters	Δρ_max_ = 0.26 e Å^−^^3^
1 restraint	Δρ_min_ = −0.48 e Å^−^^3^

### Special details

**Table:**

Geometry. All e.s.d.'s (except the e.s.d. in the dihedral angle between two l.s. planes) are estimated using the full covariance matrix. The cell e.s.d.'s are taken into account individually in the estimation of e.s.d.'s in distances, angles and torsion angles; correlations between e.s.d.'s in cell parameters are only used when they are defined by crystal symmetry. An approximate (isotropic) treatment of cell e.s.d.'s is used for estimating e.s.d.'s involving l.s. planes.
Refinement. Refinement of *F*^2^ against ALL reflections. The weighted *R*-factor *wR* and goodness of fit *S* are based on *F*^2^, conventional *R*-factors *R* are based on *F*, with *F* set to zero for negative *F*^2^. The threshold expression of *F*^2^ > σ(*F*^2^) is used only for calculating *R*-factors(gt) *etc*. and is not relevant to the choice of reflections for refinement. *R*-factors based on *F*^2^ are statistically about twice as large as those based on *F*, and *R*- factors based on ALL data will be even larger.

### Fractional atomic coordinates and isotropic or equivalent isotropic displacement parameters (Å^2^)

**Table:**

	*x*	*y*	*z*	*U*_iso_*/*U*_eq_	
Cl1	0.21684 (6)	1.16019 (5)	0.46800 (10)	0.0837 (3)	
Cl2	0.53314 (6)	0.63298 (7)	0.15637 (9)	0.0856 (3)	
N1	0.19380 (12)	0.83421 (13)	0.60855 (18)	0.0400 (4)	
N2	0.24735 (13)	0.76623 (13)	0.51905 (19)	0.0427 (4)	
O1	0.26043 (12)	0.63872 (10)	0.71199 (17)	0.0540 (4)	
C1	0.12399 (14)	1.00751 (15)	0.6282 (2)	0.0385 (5)	
C2	0.12993 (16)	1.11524 (18)	0.5925 (3)	0.0490 (5)	
C3	0.06753 (19)	1.18886 (19)	0.6553 (3)	0.0611 (6)	
H3	0.0727	1.2605	0.6301	0.073*	
C4	−0.0017 (2)	1.1565 (2)	0.7544 (3)	0.0632 (7)	
H4	−0.0443	1.2059	0.7952	0.076*	
C5	−0.00819 (18)	1.0505 (2)	0.7936 (3)	0.0587 (6)	
H5	−0.0545	1.0284	0.8622	0.070*	
C6	0.05422 (16)	0.97729 (17)	0.7309 (2)	0.0481 (5)	
H6	0.0494	0.9060	0.7581	0.058*	
C7	0.18546 (15)	0.92841 (16)	0.5543 (2)	0.0421 (5)	
H7	0.2187	0.9475	0.4668	0.050*	
C8	0.27659 (15)	0.66994 (15)	0.5784 (2)	0.0395 (5)	
C9	0.33107 (14)	0.60210 (16)	0.4702 (2)	0.0385 (5)	
C10	0.39762 (14)	0.64550 (17)	0.3718 (2)	0.0434 (5)	
H10	0.4085	0.7185	0.3701	0.052*	
C11	0.44732 (15)	0.57875 (19)	0.2767 (2)	0.0491 (5)	
C12	0.43214 (18)	0.4709 (2)	0.2764 (3)	0.0604 (6)	
H12	0.4660	0.4271	0.2108	0.073*	
C13	0.36611 (18)	0.4287 (2)	0.3747 (3)	0.0597 (6)	
H13	0.3549	0.3558	0.3750	0.072*	
C14	0.31610 (16)	0.49373 (16)	0.4730 (2)	0.0480 (5)	
H14	0.2726	0.4645	0.5406	0.058*	
H2	0.2558 (19)	0.784 (2)	0.4177 (15)	0.080*	

### Atomic displacement parameters (Å^2^)

**Table:**

	*U*^11^	*U*^22^	*U*^33^	*U*^12^	*U*^13^	*U*^23^
Cl1	0.0797 (5)	0.0687 (5)	0.1097 (6)	0.0039 (3)	0.0387 (4)	0.0327 (4)
Cl2	0.0801 (5)	0.1113 (6)	0.0754 (5)	0.0101 (4)	0.0480 (4)	0.0065 (4)
N1	0.0445 (9)	0.0439 (10)	0.0329 (9)	0.0052 (7)	0.0100 (7)	−0.0031 (7)
N2	0.0552 (10)	0.0449 (10)	0.0309 (9)	0.0109 (8)	0.0159 (8)	0.0019 (7)
O1	0.0810 (11)	0.0488 (9)	0.0364 (8)	0.0073 (7)	0.0237 (7)	0.0049 (6)
C1	0.0390 (10)	0.0428 (12)	0.0332 (10)	0.0044 (8)	0.0028 (8)	−0.0010 (8)
C2	0.0436 (11)	0.0519 (14)	0.0509 (13)	0.0030 (10)	0.0043 (9)	0.0033 (10)
C3	0.0637 (14)	0.0438 (13)	0.0743 (17)	0.0098 (11)	0.0037 (13)	−0.0032 (12)
C4	0.0640 (15)	0.0614 (16)	0.0644 (16)	0.0179 (12)	0.0092 (13)	−0.0153 (12)
C5	0.0548 (13)	0.0700 (16)	0.0540 (14)	0.0091 (12)	0.0177 (11)	−0.0036 (12)
C6	0.0507 (12)	0.0467 (12)	0.0482 (13)	0.0017 (10)	0.0116 (10)	−0.0008 (10)
C7	0.0435 (11)	0.0482 (12)	0.0357 (11)	0.0037 (9)	0.0098 (8)	0.0013 (9)
C8	0.0427 (11)	0.0438 (12)	0.0331 (11)	0.0010 (9)	0.0092 (8)	0.0002 (9)
C9	0.0376 (10)	0.0452 (12)	0.0323 (10)	0.0052 (8)	0.0033 (8)	−0.0015 (8)
C10	0.0449 (11)	0.0507 (13)	0.0351 (11)	0.0046 (9)	0.0072 (9)	−0.0016 (9)
C11	0.0445 (11)	0.0647 (15)	0.0400 (12)	0.0093 (10)	0.0122 (9)	−0.0011 (10)
C12	0.0610 (14)	0.0699 (17)	0.0511 (14)	0.0208 (12)	0.0102 (12)	−0.0151 (12)
C13	0.0675 (15)	0.0473 (14)	0.0640 (16)	0.0079 (11)	0.0077 (12)	−0.0082 (11)
C14	0.0502 (12)	0.0478 (13)	0.0468 (13)	0.0037 (10)	0.0089 (10)	0.0005 (10)

### Geometric parameters (Å, °)

**Table:**

Cl1—C2	1.735 (2)	C5—C6	1.380 (3)
Cl2—C11	1.743 (2)	C5—H5	0.9300
N1—C7	1.269 (2)	C6—H6	0.9300
N1—N2	1.386 (2)	C7—H7	0.9300
N2—C8	1.346 (2)	C8—C9	1.491 (3)
N2—H2	0.895 (10)	C9—C14	1.379 (3)
O1—C8	1.227 (2)	C9—C10	1.387 (3)
C1—C6	1.387 (3)	C10—C11	1.377 (3)
C1—C2	1.393 (3)	C10—H10	0.9300
C1—C7	1.467 (3)	C11—C12	1.372 (3)
C2—C3	1.384 (3)	C12—C13	1.375 (3)
C3—C4	1.368 (4)	C12—H12	0.9300
C3—H3	0.9300	C13—C14	1.384 (3)
C4—C5	1.379 (3)	C13—H13	0.9300
C4—H4	0.9300	C14—H14	0.9300
			
C7—N1—N2	114.25 (16)	N1—C7—H7	119.6
C8—N2—N1	119.83 (15)	C1—C7—H7	119.6
C8—N2—H2	120.5 (17)	O1—C8—N2	123.24 (18)
N1—N2—H2	119.3 (17)	O1—C8—C9	121.38 (18)
C6—C1—C2	117.34 (18)	N2—C8—C9	115.39 (16)
C6—C1—C7	121.13 (18)	C14—C9—C10	120.11 (19)
C2—C1—C7	121.46 (19)	C14—C9—C8	118.42 (18)
C3—C2—C1	121.2 (2)	C10—C9—C8	121.45 (18)
C3—C2—Cl1	118.42 (19)	C11—C10—C9	118.9 (2)
C1—C2—Cl1	120.35 (16)	C11—C10—H10	120.6
C4—C3—C2	120.1 (2)	C9—C10—H10	120.6
C4—C3—H3	119.9	C12—C11—C10	121.7 (2)
C2—C3—H3	119.9	C12—C11—Cl2	119.40 (17)
C3—C4—C5	119.9 (2)	C10—C11—Cl2	118.91 (18)
C3—C4—H4	120.1	C11—C12—C13	118.9 (2)
C5—C4—H4	120.1	C11—C12—H12	120.5
C4—C5—C6	119.9 (2)	C13—C12—H12	120.5
C4—C5—H5	120.1	C12—C13—C14	120.7 (2)
C6—C5—H5	120.1	C12—C13—H13	119.7
C5—C6—C1	121.6 (2)	C14—C13—H13	119.7
C5—C6—H6	119.2	C9—C14—C13	119.7 (2)
C1—C6—H6	119.2	C9—C14—H14	120.1
N1—C7—C1	120.73 (18)	C13—C14—H14	120.1

### Hydrogen-bond geometry (Å, °)

**Table:**

*D*—H···*A*	*D*—H	H···*A*	*D*···*A*	*D*—H···*A*
N2—H2···O1^i^	0.90 (1)	1.98 (1)	2.854 (2)	164 (2)
C7—H7···O1^i^	0.93	2.51	3.254 (2)	137 (2)

Symmetry codes: (i) *x*, −*y*+3/2, *z*−1/2.
